# Systematic Error in Hippocampal Volume Asymmetry Measurement is Minimal with a Manual Segmentation Protocol

**DOI:** 10.3389/fnins.2012.00179

**Published:** 2012-12-13

**Authors:** Baxter P. Rogers, Julia M. Sheffield, Andrew S. Luksik, Stephan Heckers

**Affiliations:** ^1^Department of Radiology and Radiological Sciences, Vanderbilt UniversityNashville, TN, USA; ^2^Department of Biomedical Engineering, Vanderbilt UniversityNashville, TN, USA; ^3^Department of Psychiatry, Vanderbilt UniversityNashville, TN, USA

**Keywords:** hippocampus, asymmetry, MRI volumetry, segmentation, asymmetry bias

## Abstract

Hemispheric asymmetry of hippocampal volume is a common finding that has biological relevance, including associations with dementia and cognitive performance. However, a recent study has reported the possibility of systematic error in measurements of hippocampal asymmetry by magnetic resonance volumetry. We manually traced the volumes of the anterior and posterior hippocampus in 40 healthy people to measure systematic error related to image orientation. We found a bias due to the side of the screen on which the hippocampus was viewed, such that hippocampal volume was larger when traced on the left side of the screen than when traced on the right (*p* = 0.05). However, this bias was smaller than the anatomical right > left asymmetry of the anterior hippocampus. We found right > left asymmetry of hippocampal volume regardless of image presentation (radiological versus neurological). We conclude that manual segmentation protocols can minimize the effect of image orientation in the study of hippocampal volume asymmetry, but our confirmation that such bias exists suggests strategies to avoid it in future studies.

## Introduction

The hippocampus is important for memory formation (Eichenbaum, 2004) and is affected in aging and disease (Raz et al., 2004a,b; Sperling, 2007; Mechanic-Hamilton et al., 2009; Reitz, 2009; Heckers and Konradi, 2010). Hippocampal volume, in particular, has been linked to cognition (Van Petten, 2004).

Hippocampal volume asymmetry is a common finding, and there is increasing evidence that it has biological relevance. *In vivo* MRI volumetry consistently shows that the right hippocampus is larger than the left (Pedraza et al., 2004). Hippocampal volume asymmetry is associated with dementia (Wolf et al., 2001), may be increased in mild cognitive impairment (Shi et al., 2009), and correlates with cognitive performance, particularly verbal learning, and fluency (Woolard and Heckers, 2012).

Hippocampal volume measurements may be affected by systematic error in segmentation procedures. This includes a dependence on MR imaging parameters (Pedraza et al., 2004). Additionally, there is a right/left visual perceptive bias (Jewell and McCourt, 2000; Guo et al., 2009) that may cause estimated volumes to depend on the orientation of the image as presented to a human rater. Recently Maltbie et al. (2012) reported that volume asymmetry of the hippocampus is nearly inverted if images are presented in mirrored fashion (12% greater versus 8% smaller right hippocampal volumes). They used a semi-automated procedure to measure hippocampal volume (Csernansky et al., 1998) and concluded that the user interaction (i.e., placing landmarks and manually adjusting a surface template to optimize the hippocampal segmentation) is responsible for the asymmetric bias.

We have recently shown that volume asymmetry is limited to the anterior hippocampus and that this localized asymmetry is correlated with cognitive performance (Woolard and Heckers, 2012). In contrast with Maltbie et al. (2012), we used a fully manual segmentation protocol derived from an earlier study (Pruessner et al., 2000). Our protocol involved the manual identification of the edges of the hippocampus on coronal and sagittal sections. Here we measured hemispheric asymmetry and bias related to image orientation for volumes of anterior and posterior hippocampus in order to estimate any error due to visual perceptive bias in a manual segmentation protocol.

## Materials and Methods

### Participants

Study subjects were 40 healthy control volunteers with a structural MRI scan and a completed Structured Clinical Interview of the DSM IV-TR (First et al., 1996), randomly selected from a large repository study (Psychiatric Genotype/Phenotype Project Repository). None reported having any major medical or neurologic illness, Axis I psychiatric disorder or psychotropic medication use, including alcohol or substance abuse or dependence (see Table 1 for demographics). Participants gave written informed consent under a protocol approved by the Vanderbilt University Institutional Review Board, Nashville, TN, USA.

**Table 1:** **Participant demographics for the sample of 40**.

Age	27 (14), 22–37
Sex	23 male, 17 female
Race	27 white, 10 black, 3 other
Handedness	36 right, 4 left
IQ (WTAR)	112 (20), 100–119

*Age and IQ are reported as Median (IQR), Q1–Q3*.

### MR image acquisition

Images were collected on a 3 T Philips Intera Achieva scanner located at the Vanderbilt University Institute of Imaging Science. A fiducial marker (vitamin E capsule) was placed on the forehead of every participant to unambiguously indicate the right hemisphere in the MR images. A high-resolution T1-weighted fast field echo (FFE) structural scan (170 sagittal slices, matrix 256 × 256, 1.0 mm isotropic voxel size, TR 8.0 ms, TE 3.7 ms) was acquired as part of a larger imaging protocol. The T1 scan volume was aligned so that the anterior-posterior image axis was approximately parallel to the line between anterior and posterior commissures.

### Manual segmentation of hippocampus

Structural images for each person were duplicated and one duplicate was flipped through the left-right axis, resulting in a total of 80 distinct brain images. The brain images were then randomized and blinded by one author (Baxter P. Rogers), such that the fiducial marker was no longer visible and the participant ID and image orientation were not indicated. Manual segmentation of both hippocampi was then performed on both the original and flipped images by one right handed, blinded rater (Julia M. Sheffield).

Manual segmentation used a slightly modified version of a previously established protocol described in detail elsewhere (Pruessner et al., 2000; Woolard and Heckers, 2012). Intra-class correlation between repeated measurements by the same rater under this protocol was 0.88–0.89 for posterior and 0.96–0.98 for anterior volumes in 16 randomly selected brains (Woolard and Heckers, 2012). All segmentations were performed using 3D Slicer (versions 3.4 and 3.6), a free open source software package used for visualization and medical image computing (Pieper et al., 2004). 3D Slicer allows for simultaneous viewing of MR images in three orthogonal orientations and automatically calculates the volumes of manually segmented structures.

Segmentation was performed in native space (i.e., on non-normalized, non-warped images). Each hippocampus was outlined in the sagittal view on each 1 mm slice, proceeding from lateral to medial. Once the hippocampus was fully outlined in the sagittal orientation, the outline was further refined using the Pruessner protocol in the coronal orientation (Pruessner et al., 2000). Under this protocol, the rater began with the most posterior section and proceeded anteriorly until the hippocampus receded under the amygdala. Although segmentation of the hippocampus generally followed the Pruessner protocol, there were a few deviations (Woolard and Heckers, 2012). First, the arbitrary borders used to separate the hippocampal tail from surrounding regions (i.e., fasciolar gyrus and Andreas Retzius gyrus) described within the Pruessner protocol were not used. Instead we visually approximated the correct gray matter boundary of the hippocampal tail, with the knowledge that we may have included portions of the fasciolar gyrus and Andreas Retzius gyrus. Second, rather than drawing a 45° line from the inferior part of the hippocampal body medially to the quadrigeminal cistern, we followed the contours of the underlying parahippocampal gyrus to the quadrigeminal cistern. Finally, the Pruessner protocol separates the hippocampus into the head, body, and tail. Instead, we separated hippocampus into only two distinct regions, i.e., anterior (uncus) and posterior (body, tail). The anterior/posterior boundary was identified in the coronal orientation by the presence of more than one cut through the hippocampus (Duvernoy, 1988). This anterior-posterior demarcation was then verified in the sagittal view, resulting in four distinct regions of the hippocampus: right anterior, right posterior, left anterior, and left posterior. The entire segmentation protocol was performed first for the hippocampus on the right side of the image, then repeated for the hippocampus on the left side of the image.

### Asymmetry analysis

Volumes were calculated in 3D Slicer for each of the four regions of the hippocampus. This was done on all 80 scans, yielding hippocampal volumes for our 40 subjects as traced in both the radiological and neurological presentations. “Radiological” indicates that asymmetry was calculated from the measured volume of the left hippocampus when it was shown on the right side of the screen in coronal section and of the right hippocampus when it was shown on the left side of the screen; vice versa for “neurological.” Asymmetry was measured as the right-minus-left volume difference as a fraction of the mean volume:

$$A=2\frac{V_{\text{R}}-V_{\text{L}}}{V_{\text{R}}+V_{\text{L}}}$$

where *V*_R_ and *V*_L_ are the anatomical right and left hippocampal volumes. This formula provided an index of right-left asymmetry in the hippocampus and allowed us to compare this asymmetry within subjects for radiological and neurological image orientation.

### Statistical analysis

Raw measures of regional volume were analyzed using a repeated measures analysis of variance (ANOVA), with anatomical hemisphere (right or left), hippocampal image orientation (right or left side of screen image in coronal section), and hippocampal region (anterior or posterior) as within-subject factors. This allowed us to test directly for a visual perceptive bias when tracing coronal images of the hippocampus on the right or left side of the computer screen (image orientation). In addition, to assess the impact of image presentation (radiological versus neurological) on measured hippocampal asymmetry, we used paired *t*-tests to compare the respective asymmetry indices.

## Results

**Figure 1:**
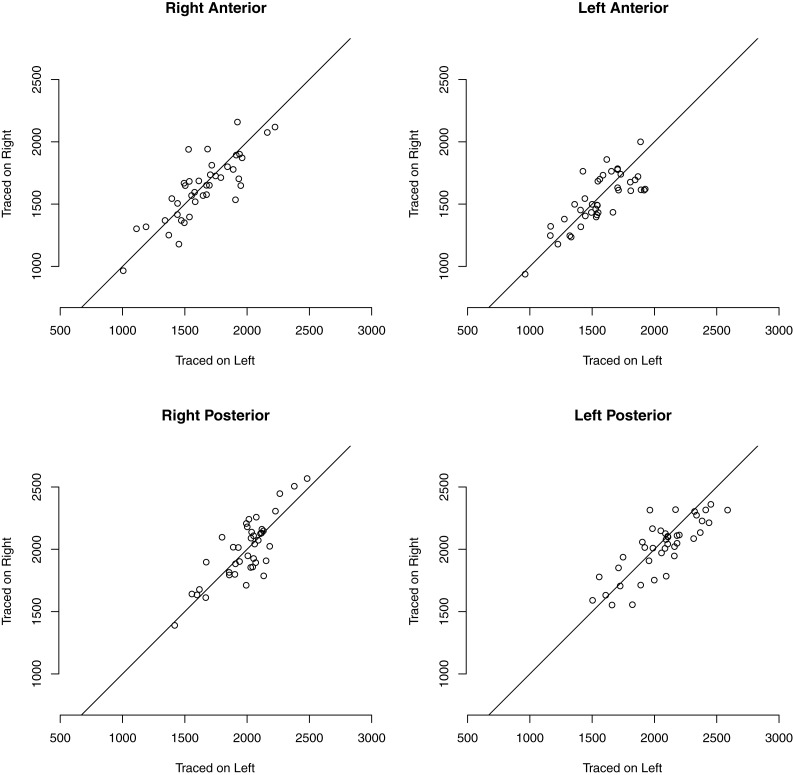
**Minimal discrepancy between measured volumes of right-appearing and left-appearing hippocampi**. Points show measured hippocampal volume when the structure was presented on the right side of the image in coronal section, versus when presented on the left. Systematic error would appear as a shift away from the unity line. Statistical analysis in Section “Results.”

We measured the dependence of raw hippocampal volumes on image orientation during tracing. Figure 1 shows the volume for each of the four hippocampal regions when traced on the right side of the screen image versus the volume when traced on the left. There was a main effect of image orientation – 17 mm^3^, *F*(1, 39) = 4.21, *p* = 0.05 – indicating a tracing bias due to the side of the screen on which the hippocampus was viewed, such that a hippocampus was measured to be larger when traced on the left side of the screen than when traced on the right. Other main effects were anatomical hemisphere [right greater than left, *F*(1, 39) = 1.54, *p* = 0.22] and hippocampal region [posterior greater than anterior, *F*(1, 39) = 88.95, *p* < 0.0001]. The anterior hippocampus was larger on the right by 88 mm^3^, while the posterior hippocampus was larger on the left by 48 mm^3^, a two-way interaction between anatomical hemisphere and hippocampal region: *F*(1, 39) = 9.59, *p* = 0.004. There was no evidence for two-way interactions between anatomical hemisphere and image orientation – *F*(1, 39) = 0.94, *p* = 0.34 – or between hippocampal region and image orientation: *F*(1, 39) = 0, *p* = 0.99. There was no evidence for a three-way interaction between anatomical hemisphere, hippocampal region, and image orientation: *F*(1, 39) = 0.78, *p* = 0.38. For mean volumes of each region, see Table 2.

**Table 2:** **Mean volumes of hippocampal regions in mm^3^**.

	Anterior		Posterior	
	Left hipp	Right hipp	Left hipp	Right hipp
Traced on right	1535	1628	2017	1995
Traced on left	1558	1640	2060	1986
Traced R versus L	*T* = −1.0, *p* = 0.34	*T* = −0.5, *p* = 0.62	*T* = −1.8, *p* = 0.08	*T* = 0.4, *p* = 0.70

*“Traced on …” indicates image orientation – the side of the screen image where the hippocampus was presented in coronal section. Volumes when traced on right versus traced on left were compared directly with paired *t*-tests*.

**Figure 2:**
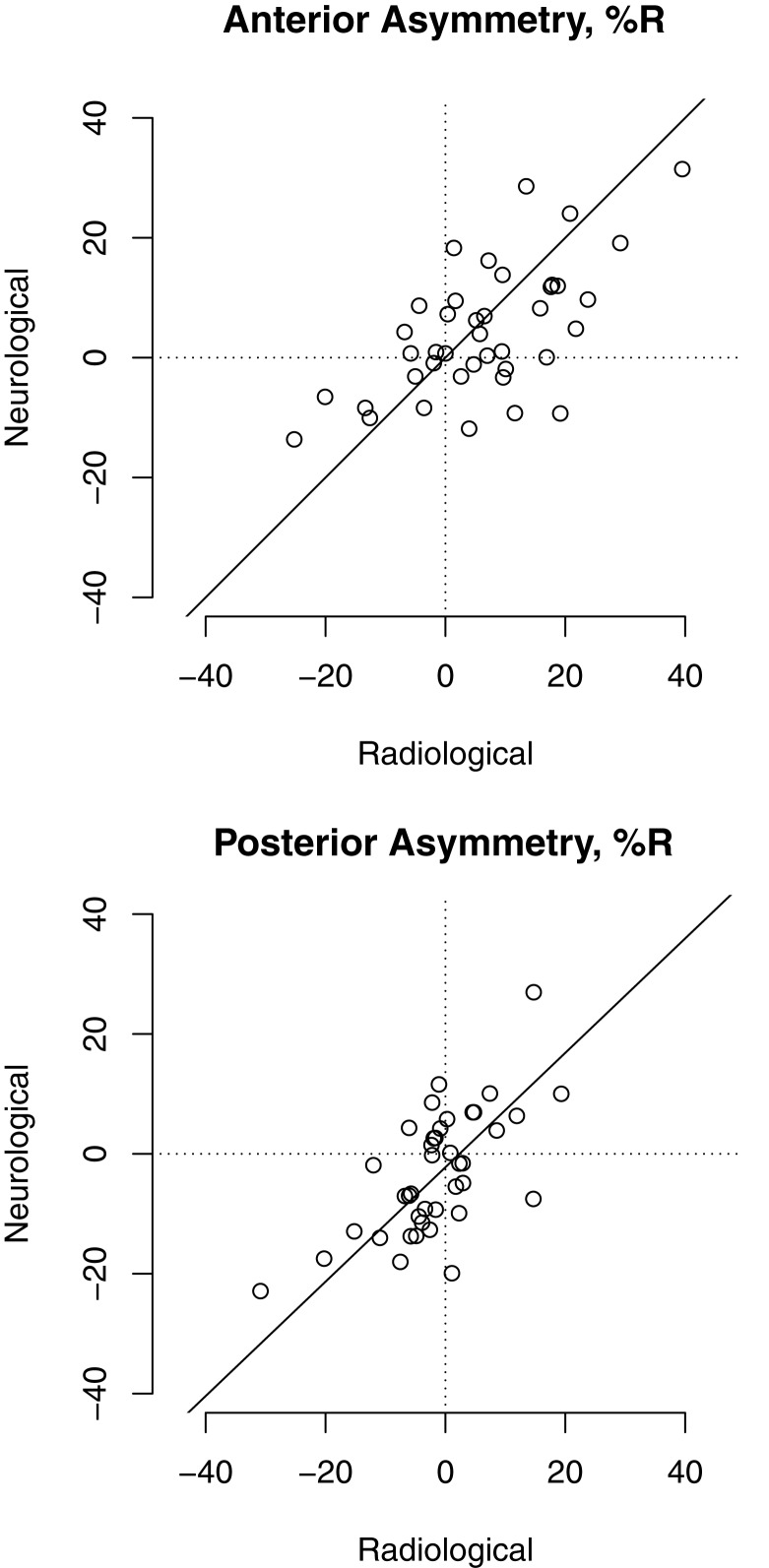
**Minimal discrepancy between measured asymmetry indices calculated from images in radiological orientation (right-on-left) versus neurological orientation (right-on-right)**. Systematic error would appear as a shift away from the unity line. Statistical analysis in Table 3.

We also measured the effect of image presentation on asymmetry indices. Figure 2 shows asymmetry calculated from hippocampi traced in radiological presentation (left hippocampus traced on the right side of the image and right hippocampus traced on the left) versus neurological presentation (the opposite). Points cluster closer around the unity line compared with the large bias evident in Maltbie et al. (2012), Figure 2, second panel. Quantitative analysis is shown in Table 3; a right-greater-than-left asymmetry was present and statistically significant in anterior hippocampus regardless of image presentation, and there was not a statistically significant difference between the two orientations in the estimate of asymmetry index.

**Table 3:** **Measured asymmetry index did not depend strongly on image presentation**.

	Radiological presentation	Neurological presentation	Difference (rad – neuro)
Anterior	6.3% (*p* = 0.004)	4.2% (*p* = 0.018)	2.1% (*p* = 0.23)
Posterior	−1.5% (*p* = 0.31)	−3.2% (*p* = 0.06)	1.7% (*p* = 0.21)

A right-greater-than-left asymmetry was found in anterior hippocampus but was marginal or absent in posterior hippocampus for both radiological and neurological presentations. “Radiological” indicates that asymmetry was calculated from the measured volume of the left hippocampus when it was presented on the right side of the screen in coronal section and of the right hippocampus when it was presented on the left side of the screen; vice versa for “Neurological.”

## Discussion

Our manual segmentation protocol is a reliable method for measuring the asymmetry of anterior and posterior hippocampal volume. We did observe a bias related to the orientation of the coronal sections on the screen, such that a hippocampus was measured to be larger when traced on the left side of the brain image than when traced on the right. This was reflected both in raw volumes (Table 2) and in the calculated asymmetry index (Table 3). This is consistent with the existence of a visual perceptive bias (Jewell and McCourt, 2000; Guo et al., 2009) that affects the labeling of boundaries. The bias was 19% of the estimated true anatomical asymmetry, large enough that it may be important in practice. However, in our data the observed bias was smaller in magnitude than the true anatomical asymmetry of the structure and thus did not affect our conclusions about hippocampal asymmetry, regardless of how asymmetry was calculated.

Our desire to measure the systematic error of this hippocampal volumetry protocol stemmed from a recent study of bias in asymmetry measurements in human neuroimaging studies (Maltbie et al., 2012). In a sample of two pediatric and three adult cases with various diagnoses, the authors found that hippocampal asymmetry was reliably rightwards (right greater than left) when hippocampi were segmented in the neurological presentation with the right hemisphere shown on the right side of the screen, but reliably leftwards when segmentation was performed in the opposite radiological presentation. They observed a considerably larger difference between the two presentations than we did (asymmetry index of 12% for neurological versus −8% for radiological, compared to our observation of 4 versus 6%). Also, they observed that the bias was in the opposite direction, such that the neurological presentation caused the right hippocampus to appear larger instead of smaller. The segmentation protocol of Maltbie et al. (2012) – a semi-automated procedure that relied on manually selected landmarks – was quite different from our fully manual protocol. The discrepancies between our results and theirs therefore suggest that the magnitude and direction of any visual perceptive bias depend on the details of the segmentation method as well as the orientation of the images.

For our segmentation protocol, such error was small enough that it did not substantially affect conclusions about hippocampal asymmetry. However, having independently observed the possibility of systematic error in measurements of hippocampal asymmetry, we concur with the recommendation of Maltbie et al. (2012) that the effects of any visual perceptive bias on asymmetry estimates be limited by segmenting all hippocampi on the same side of the screen image so the bias affects both hemispheres equally, or by segmenting all hippocampi on both sides of the screen image at the cost of doubling the effort required (e.g., Thompson et al., 2008).

## Conflict of Interest Statement

The authors declare that the research was conducted in the absence of any commercial or financial relationships that could be construed as a potential conflict of interest.
